# Is Late Recurrence after Radical Resection for Ampullary Carcinoma A Problem?

**DOI:** 10.1155/1995/71463

**Published:** 1995

**Authors:** Steven M. Strasberg

**Affiliations:** FRCSC Section of Hepatobiliary Pancreatic Surgery Washington University School of Medicine St. Louis Missouri USA

## Abstract

Of 36 patients with carcinoma of the ampulla of Vater who underwent surgery between 1971 and 1990, 31 had a radical operation. There was one operative death. The overall 5- and 10-year survival rates were 56 and 37 per cent respectively. Survival was significantly influenced by tumour stage (p=0.0002), lymph node sthtus (p=0.006) and the degree of differentiation of the lesion (P=0.01). Three patients developed local recurrence after local excision of the tumour. Local or hepatic recurrence was common, even 5 years after pancreatoduodenectomy (four of 18 patients who suffered relapse). Radical resection can be curative in selected patients with ampullary carcinoma but late recurrence suggests the need for careful lifelong follow-up.


*HPB Surgery*, 1995, Vol. 9, pp. 59–60
Reprints available directly from the publisher
Photocopying permitted by license only

© 1995 OPA (Overseas Publishers Association)
Amsterdam B.V. Published in The Netherlands
by Harwood Academic Publishers GmbH
Printed in Malaysia

### HPB INTERNATIONAL

**EDITORIAL & ABSTRACTING SERVICE**

JOHN TERBLANCHE, EDITOR
Department of Surgery
Medical School · Observatory 7925
Cape Town · South Africa
Telephone -27-21-406-6232
Telefax -27-21-448-6461
Email: JTERBLAN@UCT GSH1. UCT. AC. ZA.

### IS LATE RECURRENCE AFTER RADICAL RESECTION FOR AMPULLARY CARCINOMA A PROBLEM?

### ABSTRACT

*Sperti, C., Pasquali, C., Piccoli, A., Sernagiotto, C. and Pedrazzoli, S. (1994) Radical resection for ampullary carcinoma: long-term results. British Journal of Surgery, 81: 668-671.*

**Of 36 patients with carcinoma of the ampulla of Vater who underwent surgery between 1971 and 1990, 31 had a radical operation. There was one operative death. The overall 5- and 10-year survival rates were 56 and 37 per cent respectively. Survival was significantly influenced by tumour stage (p=0.0002), lymph node status (p=0.006) and the degree of differentiation of the lesion (P=0.01). Three patients developed local recurrence after local excision of the tumour. Local or hepatic recurrence was common, even 5 years after pancreatoduodenectomy (four of 18 patients who suffered relapse). Radical resection can be curative in selected patients with ampullary carcinoma but late recurrence suggests the need for careful lifelong follow-up.**

KEY WORDS: Ampullary carcinoma pancreatoduodenectomy.

### PAPER DISCUSSION

The purpose of this study was to evaluate factors influencing prognosis after resection of ampullary carcinoma. Three factors were found to do so – disease stage, lymph node metastases, and tumor differentiation. These all were very powerful prognostic variables as no patients with stage III and IV tumor, positive nodes, or an undifferentiated tumor survived 5 years, When a multivariate analysis was done, however, none of the three variables were significant. Several other potential prognostic factors such as tumor size, jaundice, and pancreatic invasion, which have been found to be significant in other studies, were not significantly related to outcome in this analysis.

Three other interestingfindings were that local resection gave poor results, late recurrence of tumor after Whipple resection or local resection occurred more frequently than expected (4/18 patients had recurrence after 5 years), and about 40% of patients had a non-icteric presentation. There was one postoperative death and morbidity rates were in keeping with recent reports for this surgery.

There have been a number of case series of ampullary carcinoma in recent years as noted by the authors. The number of patients available for study is small, as in this series, and usually have been gathered over many years. The 36 patients in this study were accrued over 19 years, an intake of fewer than 2 patients per year. This difficulty of small numbers hounds us in many areas of surgery and its effect can certainly be seen in this literature. It leads to many problems of interpretation. The chances of obtaining representative or comparable groups are reduced when only 36 patients are studied. In this analysis for instance 40% of patients were not icteric, a value about double that in other series. As non icteric patients seem to have a better prognosis[1], the group presented may not be representative of patients with ampullary carcinoma in general. Furthermore, when patients are gathered over such a long period as 19 years, one necessarily asks whether one is dealing with the same disease at the beginning and end of the period. This may be so biologically, but since diagnosis and treatment methods change radically over the treatment period, as in this instance, one hardly has a stable group and this definitely affects the confidence with which one regards the results.

Another effect of small numbers is that when one looks at risk factors for outcome, different series will probably identify different risk factors, since outcome in only a few patients can affect results. In very small series only overwhelming factors such as the all or none variables found in this study are likely to be identified. Even then the numbers were too small to permit sorting by multivariate analysis to determine which of these is or are the true independent variable(s). Small numbers also explain how these authors can conclude that local excision provides poor results while others conclude that it has a reasonably good outcome. For instance Newman et al reported 43% five year survival after local excision of carcinoma of the ampulla in 9 patients[2] and Wise reported a 38% five year survival in a similar group of 8 patients[3]. The point is not that the latter outcomes are more accurate than that reported in this paper by Sperti *et al.* but that with such small numbers it is difficult to establish an accurate figure. Thus also when Sperti *et al.* conclude that late recurrence is a relatively common event, do we accept this or ask if it is an observation out at the end of the bell curve which affected this group of patients but would not be seen if several hundred patients had been studied. Let me be clear that these criticisms are not of this particular paper. The results of treatment were very good, the report is clear and the evaluation by proper statistical methods. Their review of the literature is fair and complete. The problem is a general one. Case series are a limited way of developing new knowledge and small case series are even more limited. We have been using this approach for about 100 hundred years. As we approach a new century perhaps we should ask how surgeons can cooperate to produce large prospective studies in instances such as these, in order to obtain conclusive information.

### REFERENCES

1. Yamaguchi, K., Enjoji, M. and Kitamura, K. (1990) Non-icteric ampullary carcinoma with a favorable prognosis. *Am. J. Gastroent.*, **85**, 994–999.
2. Newman, R.J. and Pittam, M.R. (1982). Local excision in the treatment of carcinoma of the ampuila of Vater. J. *Coll. Surg., Edinb*, **27**, 154–157.
3. Wise, L., Pizzimbuono, C. and Dehner, L.P. (1976) Peri-ampullary cancer. A histopathologic study in sixty-two patients. *Am. J. Surg.*, **131**,141–148.

Steven M Strasberg, MD, FRCSC
Section of Hepatobiliary Pancreatic Surgery
Washington University School of Medicine
St Louis
Missouri
USA

